# Perioperative results of mechanical valve implantantation in patients with acquired valve disease

**DOI:** 10.1186/1749-8090-8-S1-O108

**Published:** 2013-09-11

**Authors:** J Konstanty-Kalandyk, J Piatek, P Babiarczyk, M Medrzycki, L Janik, J Sadowski

**Affiliations:** 1 Department of Cardiovacular Surgery and Transplantology, John Paul II Hospital, Cracow, Poland

## Background

The implantation of an mechanical valve in many cases remains the best treatment option for acquired valve disease. Mechanical valves, despite necessity of chronic anticoagulation, has excellent durability and favorable hemodynamic parameters. The aim of our study was to evaluate the perioperative mortality in patients undergoing implantation of a mechanical valve and identifying risk factors of death.

## Methods

1500 patients underwent mechanical valve replacement. The analysis included 960 after aortic (AVR), 455 mitral (MVR) valve surgery. Mean age in AVR was 58.7 ± 13.0 years and 59.4 ± 9.0 years in MVR. Over 70 years of life in AVR was 22% and 13% in MVR.

## Results

After mechanical aortic valve perioperative mortality was 1.95% (stenosis - 1.9%, insufficiency - 2.4%) and after mechanical mitral valve was 1.97% (stenosis - 1.6%, insufficiency- 2.75%). Mortality in the age groups <50 years, 50-60 years, 60-70 years and > 70 years amounted for mechanical aortic valve 1.6%, 1.0%, 1.7% and 3.2% and for mechanical mitral valve 1, 8%, 2.8%, 1.8% and 0% respectively. In patients with aortic stenosis greatest mortality was observed after 70 years (3.0%), similarly in aortic regurgitation (> 70 years - 8.3%). In patients with mitral stenosis greatest mortality was observed in the group <50 years (2.9%) and in the case of regurgitation greatest mortality was observed in group aged 50-60 years (6.6%). Risk factor for mortality in patients undergoing implantation of a mechanical aortic valve was age, severity of symptoms (NYHA), and chronic lung disease, and mechanical mitral valve - symptoms (NYHA) and chronic heart failure.

## Conclusions

Implantation of artificial aortic or mitral valve is associated with a low risk of perioperative death. In the case of the aortic valve mortality mainly grows with the age of the patient and in the case of the mitral valve depends on the development and severity of symptoms of cardiac failure.

